# An online global database of Hemiptera-Phytoplasma-Plant biological interactions

**DOI:** 10.3897/BDJ.7.e32910

**Published:** 2019-02-26

**Authors:** Valeria Trivellone

**Affiliations:** 1 Illinois Natural History Survey, University of Illinois, Champaign, Illinois, United States of America Illinois Natural History Survey, University of Illinois Champaign, Illinois United States of America

**Keywords:** biological relationships dataset, host plants, leafhoppers, planthoppers, plant pathogens

## Abstract

**Background:**

Phytoplasmas are phloem-limited plant pathogenic bacteria in the class Mollicutes transmitted by sap-feeding insect vectors of the Order Hemiptera. Vectors still have not yet been identified for about half of the 33 known phytoplasma groups and this has greatly hindered efforts to control the spread of diseases affecting important crops. Extensive gaps of knowledge on actual phytoplasma vectors and on the plant disease epidemiology prevent our understanding of the basic underlying biological mechanisms that facilitate interactions between insects, phytoplasmas and their host plants.

**New information:**

This paper presents a complete online database of Hemiptera-Phytoplasma-Plant (HPP) biological interactions worldwide, searchable via an online interface. The raw data are available through Zenodo at https://doi.org/10.5281/zenodo.2532738. The online database search interface was created using the 3I software (Dmitriev 2006) which enhances data usability by providing a customised web interface (http://trivellone.speciesfile.org/) that provides an overview of the recorded biological interactions and ability to discover particular interactions by searching for one or more phytoplasma, insect or plant taxa. The database will facilitate synthesis of all available and relevant data on the observed associations between phytoplasmas and their insect and plant hosts and will provide useful data to generate and test ecological and evolutionary hypotheses.

## Introduction

Phytoplasmas are a group of cell wall-less bacteria with small genome sizes (530-2220 kb) belonging to the class Mollicutes. They are obligate parasites of plant phloem tissue and are transmitted from plant to plant by sap-feeding insect vectors of the order Hemiptera (Weintraub and Beanland 2006). They are responsible for hundreds of plant diseases worldwide, causing serious damage to a variety of cultivated and wild plants (Bertaccini et al. 2014). Phytoplasmas were observed for the first time in 1967 (Doi et al. 1967) and only recently classified based on phylogenetic analysis of 16S rRNA and ribosomal protein (rp) gene sequences, revealing their diversity and phylogenetic position (Lee et al. 2000; IRPCM, Phytoplasma/Spiroplasma Working Team - Phytoplasma Taxonomy Group 2004). To date, 33 groups and more than 120 subgroups have been recognised based on 16Sr genetic data and 44 taxa have been given the provisional taxonomic status of ‘*Candidatus*’ (Harrison et al. 2018), denoting not-yet cultured bacteria. As phytoplasmas do not satisfy the four Koch postulates (i.e. four criteria used to determine if an organism is the causal agent of a disease), indirect biological evidence, such as electron microscopy observation, phytoplasma and symptom elimination after tetracycline treatments (Ishiie et al. 1967), insect and dodder transmission, has confirmed association of phytoplasmas with numerous plant diseases worldwide (Bertaccini 2007; Hogenhout et al. 2008).

The only known phytoplasma vectors are hemipteran insects belonging to the suborder Auchenorrhyncha (Fulgoromorpha and Cicadomorpha) and Sternorrhyncha (family Psyllidae). An insect acquires the phytoplasma from an infected plant, then the phytoplasma must overcome two major barriers (insect gut and salivary glands cells) in order to reach the saliva for subsequent introduction into another plants' phloem (Ammar and Hogenhout 2006). An insect species is qualified to be a vector if it is competent to transmit the pathogens (vector competence) and this condition is related to the possibility of the phytoplasma to overcome the above-mentioned barriers. The screening of hemipteran species to confirm their vector competence is usually performed using traditional laboratory methods that are extremely time-consuming and expensive and, in general, have only been performed in cases where the pathogen causes severe economic losses to agriculturally important plants. As a consequence, basic knowledge of insect vector ecology and epidemiology of the plant diseases caused by phytoplasmas remains highly incomplete and vectors of many phytoplasmas still have not been identified. In order to integrate and organise data available from disparate sources, improve knowledge of global patterns of Hemiptera, phytoplasma and plant relationships and facilitate research, an open source database platform was created. This paper presents a complete searchable online database of Hemiptera-Phytoplasma-Plant (HPP) biological interactions worldwide. The database has large potential impacts on a variety of research disciplines ranging from systematics to invasive species ecology and vector biology. It will facilitate synthesis of all available and relevant data on Hemiptera-Phytoplasma-Plant associations and testing of ecological and evolutionary hypotheses.

## General description

### Purpose

The main aim of the HPP database is to facilitate storage, retrieval and dissemination of data on Hemiptera-Phytoplasma-Plant interactions published in literature. It provides an up-to-date record of all pairwise associations recorded.

### Additional information

The entire database of HPP biological interactions (raw-HPP) is available for download as tab-delimited text at Zenodo at https://doi.org/10.5281/zenodo.2532738. The same data are accessible via an online search interface created using 3I (Dmitriev 2006), a cyberinfrastructure originally designed to serve as a workbench to create taxonomic databases and identification keys. 3I software was customised here to incorporate tools which organise and summarise data on Hemiptera- and Plant-Phytoplasma biological relationships reported worldwide. It also incorporates a rigorous nomenclature and classification for all taxa included. The raw-HPP dataset represents the database back end, also accessible via the customised 3I web interface providing user-friendly online access to the data (3I-HPP). The 3I-HPP searchable interface enhances data usability by providing an overview of the reshaped biological interactions data and it can be accessed at http://trivellone.speciesfile.org/.

At present, the database contains 1860 records of plant-phytoplasma pairwise associations and 968 records of insect-phytoplasma pairwise associations. Priority is given to the first record of association between a 16Sr phytoplasma group or subgroup and a host (plant or insect vector) reported for each country. The 3I-HPP search page includes three navigation links, one for Hemiptera, one for phytoplasmas and one for plants, to enable exploration of the taxonomic hierarchy and classification of all three major organismal groups included. A search may also be performed by entering a taxon name or other search term into the box provided. Each taxon name is displayed as a hyperlink to its own dynamically generated taxon webpage. Each taxon webpage provides an overview of the recorded interactions. An interaction record is defined as a taxon (subject of the taxon webpage) that interacts with another taxon (object cited in the overview of the investigated associations). The interactions are also displayed on a map as red dots on the geographic centroid of each country, state or province where that interaction has been documented. The place names are hyperlinked to literature references for each interaction record.

## Sampling methods

### Sampling description

Data were compiled based on an extensive literature review and are categorised according to the type of relationship (see Traits coverage section) and literature records were included only if they were considered reliable. Data from published literature were considered not suitable for inclusion in the database if no molecular analysis or inoculation tests were applied to confirm the relationship between the phytoplasma and plant or insect. Papers reporting suspected vectors (without experimental evidence) or papers which inferred a phytoplasma as causing infection on plants, based only on observations of symptoms without any clear association with a phytoplasma group or subgroup, were also excluded. The papers were retrieved mainly using Web of Science and the Funk Agricultural, Consumer and Environmental Sciences Library at the University of Urbana-Champaign (Illinois, USA). The first bibliographic search gathered more than one thousand relevant studies which were individually screened and those considered to provide sufficient evidence (molecular detection of phytoplasmas in the tissues and/or transmission trials) to support or reject a species-specific plant-phytoplasma or insect-phytoplasma relationship were retained. According to the above-mentioned criteria, 829 studies were considered reliable and included in the database, while reports based only on anecdotal evidence were discarded. All papers considered have been deposited as PDF files in a shared data storage service provided by the Illinois Natural History Survey (University of Illinois).

## Geographic coverage

### Description

**Figure 1.**
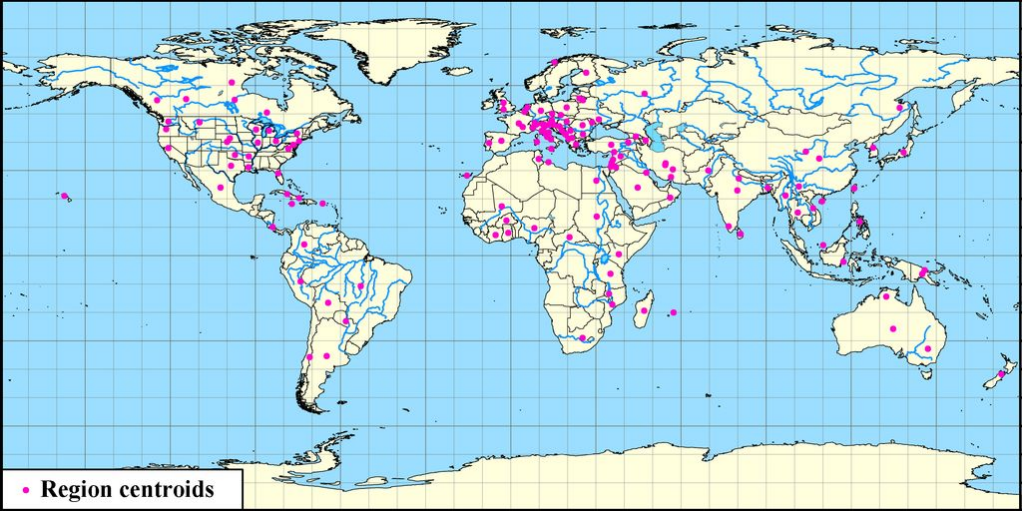
Distribution of the recorded Hemiptera- and Plant-Phytoplasma pairwise interactions (generated by http://trivellone.speciesfile.org/ web interface).

The database provides global coverage of data on documented interactions between Hemiptera, phytoplasmas and plants. In Fig. 1, the overall distribution of recorded pairwise interactions is reported.

## Taxonomic coverage

### Description

In the database, 324 taxa of Hemiptera, 44 '*Candidatus* Phytoplasma' species, 129 Phytoplasma strains and 649 plant species have been recorded.

The hemipteran species are all included in the Auchenorrhyncha and Sternorrhyncha suborders. For the first suborder, the classification follows the 3I World Auchenorrhyncha Database (Dmitriev 2003) published online; for the latter, it follows the Psyl'list database (Ouvrard 2018). Full nomenclatural information (e.g. synonymy, combinations, authorship and so on) is provided through a direct link from each 3I-HPP taxon page to 3I World Auchenorrhyncha or to the Psyl'list database.

For phytoplasmas, the classification follows the rules established by the International Code of Nomenclature of Prokaryotes (Parker et al. 2015) and additional criteria established by the Phytoplasma/Spiroplasma Working Team (IRPCM, Phytoplasma/Spiroplasma Working Team - Phytoplasma Taxonomy Group 2004, Harrison et al. 2018). All phytoplasma strains in the database are listed as *Incertae sedis* and they are classified into groups and subgroups defined based on restriction fragment length polymorphism (RFLP) analysis of Polymerase Chain Reaction (PCR)-ampliﬁed 16S rRNA gene fragments (Zhao et al. 2010).

For species recorded in the phylum of Tracheophyta (vascular plants), the classification follows the Catalogue of Life (Roskov et al. 2018).

### Taxa included

**Table:** 

Rank	Scientific Name	
order	Hemiptera	
phylum	Tracheophyta	Vascular plant
class	Mollicutes	Phytoplasma

## Traits coverage

Individual records in the raw-HPP database consist of specific pairwise interactions detected in specific countries with their literature citations. In the database, the type of relationship with phytoplasma was defined by a positive detection in an insect body or plant tissues with a consequent assignment to the phytoplasma group or subgroup (e.g. 16SrI or 16SrI A) or to a not yet characterised phytoplasma. Investigated relationships that resulted in negative detection of a phytoplasma are reported as “not detected”. The record was included if the positive or negative detection was proven using microscopic and serology-based techniques, PCR targeting the 16S–23S rRNA genes, observed or virtual RFLP fingerprinting or DNA sequencing methods (Smart et al. 1996; Namba 2011).

The 3I-HPP web interface summarises the history of the investigation for each insect and plant species. Therefore, the web interface includes 5 different categories for each taxon level, 3 for insects and 2 for plants. The category assigned to each taxon may change quickly as new investigations are carried out and new data are added to the database.

For each insect species (taxon page), one or more of the three possible categories were assigned, according to the available evidence about the capability to transmit the phytoplasma, as follows:

1. Vectorial Competence. A hemipteran species is considered a COMPETENT VECTOR for a certain phytoplasma if the capability of the latter to overcome the barriers of gut and salivary glands of the insect has been proven using classical acquisition/inoculation experiments in the laboratory or inoculation trials with caged infected specimens collected from the field. Moreover, the successful transmission of the phytoplasma to the plant has been detected on tested insects and plants using standard molecular methods, in addition to detection of clear symptoms due to phytoplasma presence in the plant. A less strict evaluation may be applied for papers published before the PCR molecular era (before 1980s), especially if the same insect species were later demonstrated to be competent vectors in the same region.
2. Positive Detection in insect. A hemipteran is considered a POTENTIAL VECTOR if the phytoplasma has been detected in its body using standard molecular methods. Status as a potential vector does not imply the ability to transmit the phytoplasma from plant to plant. Such insects may represent dead-end hosts for the phytoplasma if it is later shown that they are not competent as vectors.
3. Negative Detection in insect. A hemipteran is considered a POTENTIAL NON-VECTOR if it tested negative for the presence of phytoplasma. The more times specimens of a species have tested negative, the more likely the species is a non-vector.For each plant species, two categories were defined according to the available evidence on detection of phytoplasma in the plant phloem, as follows:
4. Positive Detection in plant. A plant is considered INFECTED BY phytoplasma if the pathogen has been successfully detected in its tissues using standard molecular methods.
5. Negative Detection in plant. A plant is considered a POTENTIAL UNSUITABLE HOST if it tested negative for the presence of phytoplasma. The more times samples of a species have tested negative, the more likely that species is an unsuitable host for the phytoplasma.

## Temporal coverage

### Notes

The relevant literature included in the database covers a time span of more than 120 years (from 1895 to date).

## Usage rights

### Use license

Оpen Data Commons Open Database License (ODbL)

## Data resources

### Data package title

Hemiptera-Phytoplasma-Plant dataset

### Resource link

https://doi.org/10.5281/zenodo.2532738

### Alternative identifiers

10.5281/zenodo.2532738

### Number of data sets

1

### Data set 1.

#### Data set name

Hemiptera-Phytoplasma-Plant dataset

#### Data format

TSV file

#### Number of columns

8

#### Download URL

https://doi.org/10.5281/zenodo.2532738

#### Data format version

v1.2.0

#### Description

This is a database of Hemiptera-Phytoplasma-Plant (HPP) biological interactions worldwide. The database contains 1860 records of plant-phytoplasma pairwise associations and 968 records of insect-phytoplasma pairwise associations. Only the earliest reported record of each specific association between a 16Sr phytoplasma subgroup and a host (plant or insect vector) is reported for each country in the database. Duplicate records confirming the same association may be added in the future.

**Data set 1.** 

Column label	Column description
record_id	record identifier
phytoplasma	Phytoplasma 16Sr groups and subgroups detected in the insect or plant host. The "not detected" entry means that phytoplasma was not detected in the host analysed and the "not characterised" entry means that it was not defined the 16Sr group for the detected phytoplasma as reported in the reference source
host_group	The group (insect or plant) to which the host belongs
host_family	Family name of the insect or plant host
host_species	Species name of the insect or plant host
country	Country where the biological association was detected or searched as reported in the reference source
reference_id	Short reference identifier for the biological interaction
full_reference	Full reference source for the biological interaction

## References

[B4975331] Ammar El-Desouky, Hogenhout Saskia, Kostas B., Miller T. (2006). Mollicutes associated with arthropods and plants. Insect Symbiosis.

[B4974843] Bertaccini Assunta (2007). Phytoplasmas: diversity, taxonomy, and epidemiology. Frontiers Bioscience.

[B4974778] Bertaccini Assunta, Duduk Bojan, Paltrinieri Samanta, Contaldo Nicoletta (2014). Phytoplasmas and Phytoplasma diseases: A severe threat to agriculture. American Journal of Plant Sciences.

[B4975506] Dmitriev D. A. 3I World Auchenorrhyncha database. http://dmitriev.speciesfile.org.

[B4974877] Dmitriev D. A. (2006). 3I, a new program for creating Internet-accessible interactive keys and taxonomic databases and its application for taxonomy of Cicadina (Homoptera). Russian Entomological Journal.

[B4974788] Doi Yoji, Teranaka Michiaki, Yora Kiyoshi, Asuyama Hidefumi (1967). Mycoplasma-or PLT group-like microorganisms found in the phloem elements of plants infected with mulberry dwarf, potato witches' broom, aster yellows, or paulownia witches' broom. Japanese Journal of Phytopathology.

[B4974818] Harrison N. A., Gundersen-Rindal Dawn, Davis R. E., May Meghan, Brown D. R. (2018). Candidatus Phytoplasma. Bergey's Manual of Systematics of Archaea and Bacteria.

[B4974865] Hogenhout Saskia A., Oshima Kenro, Ammar El-Desouky, Kakizawa Shigeyuki, Kingdom Heather N., Namba Shigetou (2008). Phytoplasmas: bacteria that manipulate plants and insects. Molecular Plant Pathology.

[B4974808] IRPCM Phytoplasma/Spiroplasma Working Team - Phytoplasma Taxonomy Group (2004). 'Candidatus Phytoplasma', a taxon for the wall-less, non-helical prokaryotes that colonize plant phloem and insects. International Journal of Systematic and Evolutionary Microbiology.

[B4974833] Ishiie T., Doi Y., Yora K., Asuyama H. (1967). Suppressive effects of antibiotics of tetracycline group on symptom development of mulberry dwarf disease. Japanese Journal of Phytopathology.

[B4974798] Lee I - M, Davis R. E., Gundersen-Rindal D. E. (2000). Phytoplasma: Phytopathogenic Mollicutes. Annual Review of Microbiology.

[B4975496] Namba Shigetou (2011). Phytoplasmas: A century of pioneering research. Journal of General Plant Pathology.

[B4975129] Ouvrard D. Psyl'list - The World Psylloidea Database.. http://www.hemiptera-databases.com/psyllist.

[B4975138] Parker C. T., Tindall B. J., Garrity G. M. (2015). International Code of Nomenclature of Prokaryotes. International Journal of Systematic and Evolutionary Microbiology.

[B4975112] Roskov Y., Ower G., Orrell T., Nicolson D., Bailly N., Kirk P. M., Bourgoin T., DeWalt R. E., Decock W., Nieukerken E. van, Zarucchi J., Penev L. Species 2000 & ITIS Catalogue of Life. www.catalogueoflife.org/col.

[B4975481] Smart C. D., Schneider B., Blomquist C. L., Guerra L. J., Harrison N. A., Ahrens U., Lorenz K. H., Seemüller E., Kirkpatrick B. C (1996). Phytoplasma-specific PCR primers based on sequences of the 16S-23S rRNA spacer region.. Applied and Environmental Microbiology.

[B4974768] Weintraub Phyllis G., Beanland LeAnn (2006). Insect vectors of phytoplasmas. Annual Review of Entomology.

[B4975204] Zhao Y., Wei W., Davis R. E., Lee I. M., Weintraub P. G., Jones P. (2010). Recent advances in 16S rRNA gene-based phytoplasma differentiation, classification and taxonomy.. Phytoplasmas: genomes, plant hosts and vectors.

